# (*E*)-*N*′-[4-(Methyl­sulfan­yl)benzyl­idene]furan-2-carbohydrazide monohydrate

**DOI:** 10.1107/S1600536810028655

**Published:** 2010-07-31

**Authors:** Yu-Feng Li, Fang-Fang Jian

**Affiliations:** a Microscale Science Institute, Department of Chemistry and Chemical Engineering, Weifang University, Weifang 261061, People’s Republic of China; b Microscale Science Institute, Weifang University, Weifang 261061, People’s Republic of China

## Abstract

In the title compound, C_13_H_12_N_2_O_2_S·H_2_O, the dihedral angle between the aromatic rings is 35.34 (19)° and an intra­molecular N—H⋯O hydrogen bond generates an *S*(5) ring. In the crystal, mol­ecules are linked by N—H⋯O and O—H⋯O hydrogen bonds, generating (001) sheets.

## Related literature

For a related structure, see: Li & Jian (2010 ▶).
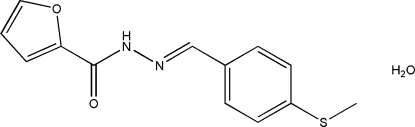


## Experimental

### 

#### Crystal data

C_13_H_12_N_2_O_2_S·H_2_O
*M* *_r_* = 278.32
Monoclinic, 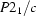

*a* = 4.7065 (9) Å
*b* = 12.142 (2) Å
*c* = 23.979 (5) Å
β = 91.96 (3)°
*V* = 1369.6 (5) Å^3^
*Z* = 4
Mo *K*α radiation
μ = 0.24 mm^−1^
*T* = 293 K
0.22 × 0.20 × 0.18 mm

#### Data collection

Bruker SMART CCD diffractometer
10766 measured reflections
2536 independent reflections
1095 reflections with *I* > 2σ(*I*)
*R*_int_ = 0.093

#### Refinement

*R*[*F* ^2^ > 2σ(*F* ^2^)] = 0.052
*wR*(*F* ^2^) = 0.172
*S* = 0.81
2536 reflections
180 parameters
H atoms treated by a mixture of independent and constrained refinement
Δρ_max_ = 0.18 e Å^−3^
Δρ_min_ = −0.25 e Å^−3^

### 

Data collection: *SMART* (Bruker, 1997 ▶); cell refinement: *SAINT* (Bruker, 1997 ▶); data reduction: *SAINT*; program(s) used to solve structure: *SHELXS97* (Sheldrick, 2008 ▶); program(s) used to refine structure: *SHELXL97* (Sheldrick, 2008 ▶); molecular graphics: *SHELXTL* (Sheldrick, 2008 ▶); software used to prepare material for publication: *SHELXTL*.

## Supplementary Material

Crystal structure: contains datablocks global, I. DOI: 10.1107/S1600536810028655/hb5554sup1.cif

Structure factors: contains datablocks I. DOI: 10.1107/S1600536810028655/hb5554Isup2.hkl

Additional supplementary materials:  crystallographic information; 3D view; checkCIF report

## Figures and Tables

**Table 1:** Hydrogen-bond geometry (Å, °)

*D*—H⋯*A*	*D*—H	H⋯*A*	*D*⋯*A*	*D*—H⋯*A*
N1—H1*B*⋯O2	0.86	2.37	2.713 (3)	104
N1—H1*B*⋯O3^i^	0.86	2.03	2.864 (4)	162
O3—H3*B*⋯O1^ii^	0.87 (5)	2.03 (6)	2.878 (4)	165 (4)
O3—H3*C*⋯O1	0.76 (8)	2.11 (8)	2.800 (4)	152 (8)

Symmetry codes: (i) 
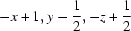
; (ii) 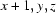.

**Fig. 1.**
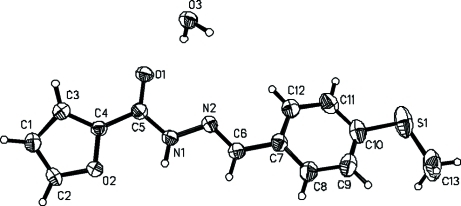
The structure of the title compound showing 30% probability displacement ellipsoids.

## supplementary crystallographic information

### Experimental

A mixture of 4-(methylthio)benzaldehyde (0.1 mol), and furan-2-carbohydrazide (0.1 mol) was stirred in refluxing ethanol (20 ml) for 2 h to afford the title compound (0.090 mol, yield 90%). Colourless blocks of the title compound were obtained by recrystallization from ethanol at room temperature.

### Refinement

H atoms were fixed geometrically and allowed to ride on their attached atoms, with C—H = 0.97 Å, and *U*_iso_ = 1.2–1.5*U*_eq_.

### Crystal data

**Table:**

C_13_H_14_N_2_O_3_S	*F*(000) = 584
*M**_r_* = 278.32	*D*_x_ = 1.350 Mg m^−^^3^
Monoclinic, *P*2_1_/*c*	Mo *K*α radiation, λ = 0.71073 Å
Hall symbol: -P 2ybc	Cell parameters from 1095 reflections
*a* = 4.7065 (9) Å	θ = 3.1–25.5°
*b* = 12.142 (2) Å	µ = 0.24 mm^−^^1^
*c* = 23.979 (5) Å	*T* = 293 K
β = 91.96 (3)°	Block, colorless
*V* = 1369.6 (5) Å^3^	0.22 × 0.20 × 0.18 mm
*Z* = 4	

### Data collection

**Table:**

Bruker SMART CCD diffractometer	1095 reflections with *I* > 2σ(*I*)
Radiation source: fine-focus sealed tube	*R*_int_ = 0.093
graphite	θ_max_ = 25.5°, θ_min_ = 3.1°
phi and ω scans	*h* = −5→5
10766 measured reflections	*k* = −14→14
2536 independent reflections	*l* = −27→29

### Refinement

**Table:**

Refinement on *F*^2^	Primary atom site location: structure-invariant direct methods
Least-squares matrix: full	Secondary atom site location: difference Fourier map
*R*[*F*^2^ > 2σ(*F*^2^)] = 0.052	Hydrogen site location: inferred from neighbouring sites
*wR*(*F*^2^) = 0.172	H atoms treated by a mixture of independent and constrained refinement
*S* = 0.81	*w* = 1/[σ^2^(*F*_o_^2^) + (0.1*P*)^2^] where *P* = (*F*_o_^2^ + 2*F*_c_^2^)/3
2536 reflections	(Δ/σ)_max_ < 0.001
180 parameters	Δρ_max_ = 0.18 e Å^−^^3^
0 restraints	Δρ_min_ = −0.25 e Å^−^^3^

### Special details

**Table:**

Geometry. All e.s.d.'s (except the e.s.d. in the dihedral angle between two l.s. planes) are estimated using the full covariance matrix. The cell e.s.d.'s are taken into account individually in the estimation of e.s.d.'s in distances, angles and torsion angles; correlations between e.s.d.'s in cell parameters are only used when they are defined by crystal symmetry. An approximate (isotropic) treatment of cell e.s.d.'s is used for estimating e.s.d.'s involving l.s. planes.
Refinement. Refinement of *F*^2^ against ALL reflections. The weighted *R*-factor *wR* and goodness of fit *S* are based on *F*^2^, conventional *R*-factors *R* are based on *F*, with *F* set to zero for negative *F*^2^. The threshold expression of *F*^2^ > σ(*F*^2^) is used only for calculating *R*-factors(gt) *etc*. and is not relevant to the choice of reflections for refinement. *R*-factors based on *F*^2^ are statistically about twice as large as those based on *F*, and *R*- factors based on ALL data will be even larger.

### Fractional atomic coordinates and isotropic or equivalent isotropic displacement parameters (Å^2^)

**Table:**

	*x*	*y*	*z*	*U*_iso_*/*U*_eq_	
C5	0.1858 (6)	0.6495 (3)	0.26977 (12)	0.0476 (8)	
N2	0.5006 (6)	0.6551 (2)	0.19538 (11)	0.0543 (7)	
C4	−0.0079 (6)	0.5827 (3)	0.30142 (12)	0.0469 (8)	
O2	−0.0668 (5)	0.47801 (18)	0.28399 (9)	0.0576 (7)	
C6	0.6577 (7)	0.5956 (3)	0.16494 (13)	0.0544 (9)	
H6A	0.6664	0.5200	0.1709	0.065*	
C1	−0.3067 (8)	0.5097 (3)	0.35996 (15)	0.0695 (11)	
H1A	−0.4258	0.5003	0.3898	0.083*	
C2	−0.2512 (7)	0.4347 (3)	0.32055 (14)	0.0647 (10)	
H2B	−0.3273	0.3641	0.3186	0.078*	
C8	1.0074 (7)	0.5792 (3)	0.09165 (14)	0.0686 (11)	
H8A	1.0343	0.5060	0.1020	0.082*	
C3	−0.1508 (8)	0.6049 (3)	0.34769 (14)	0.0620 (10)	
H3A	−0.1473	0.6705	0.3677	0.074*	
O1	0.2107 (5)	0.7491 (2)	0.27923 (10)	0.0628 (7)	
C7	0.8238 (7)	0.6447 (3)	0.12095 (12)	0.0541 (9)	
N1	0.3328 (5)	0.5959 (2)	0.23110 (10)	0.0512 (7)	
H1B	0.3227	0.5254	0.2285	0.061*	
C12	0.7927 (8)	0.7535 (3)	0.10515 (17)	0.0740 (11)	
H12A	0.6732	0.7994	0.1247	0.089*	
C11	0.9370 (9)	0.7947 (4)	0.06065 (17)	0.0823 (12)	
H11A	0.9137	0.8682	0.0507	0.099*	
C10	1.1155 (8)	0.7288 (4)	0.03055 (16)	0.0765 (12)	
C9	1.1526 (8)	0.6220 (4)	0.04680 (16)	0.0809 (13)	
H9A	1.2764	0.5771	0.0277	0.097*	
O3	0.7049 (8)	0.8706 (2)	0.30372 (13)	0.0682 (8)	
S1	1.2735 (3)	0.79024 (15)	−0.02691 (5)	0.1234 (7)	
C13	1.4598 (13)	0.6810 (6)	−0.0588 (2)	0.161 (3)	
H13A	1.5531	0.7084	−0.0910	0.241*	
H13B	1.3280	0.6242	−0.0700	0.241*	
H13C	1.5989	0.6515	−0.0327	0.241*	
H3B	0.857 (12)	0.841 (4)	0.2908 (19)	0.12 (2)*	
H3C	0.602 (17)	0.833 (7)	0.288 (3)	0.21 (4)*	

### Atomic displacement parameters (Å^2^)

**Table:**

	*U*^11^	*U*^22^	*U*^33^	*U*^12^	*U*^13^	*U*^23^
C5	0.0404 (18)	0.047 (2)	0.056 (2)	0.0052 (17)	0.0024 (15)	0.0034 (17)
N2	0.0518 (16)	0.0511 (18)	0.0606 (17)	−0.0064 (15)	0.0103 (14)	0.0021 (14)
C4	0.0471 (18)	0.041 (2)	0.0529 (18)	0.0025 (16)	0.0030 (15)	−0.0020 (15)
O2	0.0659 (14)	0.0447 (14)	0.0631 (14)	−0.0051 (12)	0.0166 (12)	−0.0027 (11)
C6	0.0518 (19)	0.052 (2)	0.059 (2)	−0.0016 (17)	0.0055 (17)	−0.0022 (17)
C1	0.082 (3)	0.063 (3)	0.065 (2)	0.002 (2)	0.023 (2)	0.007 (2)
C2	0.077 (3)	0.053 (2)	0.065 (2)	−0.011 (2)	0.021 (2)	0.0118 (19)
C8	0.060 (2)	0.080 (3)	0.066 (2)	−0.004 (2)	0.0084 (19)	−0.004 (2)
C3	0.069 (2)	0.055 (2)	0.062 (2)	0.0013 (19)	0.0133 (19)	−0.0036 (18)
O1	0.0614 (15)	0.0406 (15)	0.0878 (17)	−0.0035 (12)	0.0195 (13)	−0.0062 (12)
C7	0.0426 (18)	0.065 (3)	0.055 (2)	−0.0044 (18)	−0.0002 (16)	−0.0022 (18)
N1	0.0509 (16)	0.0406 (17)	0.0628 (16)	−0.0010 (13)	0.0091 (14)	0.0006 (13)
C12	0.073 (3)	0.066 (3)	0.083 (3)	−0.001 (2)	0.019 (2)	0.004 (2)
C11	0.083 (3)	0.076 (3)	0.089 (3)	−0.011 (2)	0.012 (2)	0.020 (2)
C10	0.059 (2)	0.109 (4)	0.061 (2)	−0.019 (3)	0.0024 (19)	0.009 (2)
C9	0.067 (3)	0.109 (4)	0.067 (3)	−0.004 (3)	0.017 (2)	−0.005 (3)
O3	0.0683 (18)	0.0448 (17)	0.0929 (19)	−0.0012 (15)	0.0231 (17)	−0.0016 (14)
S1	0.1001 (10)	0.1929 (17)	0.0779 (8)	−0.0324 (10)	0.0135 (7)	0.0432 (9)
C13	0.158 (5)	0.237 (8)	0.092 (4)	−0.087 (5)	0.068 (4)	−0.047 (4)

### Geometric parameters (Å, °)

**Table:**

C5—O1	1.235 (4)	C3—H3A	0.9300
C5—N1	1.344 (4)	C7—C12	1.380 (5)
C5—C4	1.454 (4)	N1—H1B	0.8600
N2—C6	1.280 (4)	C12—C11	1.378 (5)
N2—N1	1.385 (3)	C12—H12A	0.9300
C4—C3	1.344 (4)	C11—C10	1.381 (6)
C4—O2	1.364 (4)	C11—H11A	0.9300
O2—C2	1.360 (4)	C10—C9	1.364 (6)
C6—C7	1.461 (4)	C10—S1	1.754 (4)
C6—H6A	0.9300	C9—H9A	0.9300
C1—C2	1.344 (5)	O3—H3B	0.87 (5)
C1—C3	1.406 (5)	O3—H3C	0.75 (8)
C1—H1A	0.9300	S1—C13	1.777 (6)
C2—H2B	0.9300	C13—H13A	0.9600
C8—C7	1.384 (4)	C13—H13B	0.9600
C8—C9	1.394 (5)	C13—H13C	0.9600
C8—H8A	0.9300		
			
O1—C5—N1	123.6 (3)	C8—C7—C6	119.4 (3)
O1—C5—C4	120.5 (3)	C5—N1—N2	119.6 (3)
N1—C5—C4	115.9 (3)	C5—N1—H1B	120.2
C6—N2—N1	114.4 (3)	N2—N1—H1B	120.2
C3—C4—O2	109.7 (3)	C11—C12—C7	120.6 (4)
C3—C4—C5	131.2 (3)	C11—C12—H12A	119.7
O2—C4—C5	119.0 (3)	C7—C12—H12A	119.7
C2—O2—C4	106.9 (2)	C12—C11—C10	121.3 (4)
N2—C6—C7	121.0 (3)	C12—C11—H11A	119.4
N2—C6—H6A	119.5	C10—C11—H11A	119.4
C7—C6—H6A	119.5	C9—C10—C11	118.4 (4)
C2—C1—C3	107.1 (3)	C9—C10—S1	125.1 (4)
C2—C1—H1A	126.4	C11—C10—S1	116.5 (4)
C3—C1—H1A	126.4	C10—C9—C8	120.9 (4)
C1—C2—O2	109.6 (3)	C10—C9—H9A	119.6
C1—C2—H2B	125.2	C8—C9—H9A	119.6
O2—C2—H2B	125.2	H3B—O3—H3C	96 (6)
C7—C8—C9	120.6 (4)	C10—S1—C13	104.4 (3)
C7—C8—H8A	119.7	S1—C13—H13A	109.5
C9—C8—H8A	119.7	S1—C13—H13B	109.5
C4—C3—C1	106.7 (3)	H13A—C13—H13B	109.5
C4—C3—H3A	126.7	S1—C13—H13C	109.5
C1—C3—H3A	126.7	H13A—C13—H13C	109.5
C12—C7—C8	118.2 (3)	H13B—C13—H13C	109.5
C12—C7—C6	122.3 (3)		

### Hydrogen-bond geometry (Å, °)

**Table:**

*D*—H···*A*	*D*—H	H···*A*	*D*···*A*	*D*—H···*A*
N1—H1B···O2	0.86	2.37	2.713 (3)	104
N1—H1B···O3^i^	0.86	2.03	2.864 (4)	162
O3—H3B···O1^ii^	0.87 (5)	2.03 (6)	2.878 (4)	165 (4)
O3—H3C···O1	0.76 (8)	2.11 (8)	2.800 (4)	152 (8)

Symmetry codes: (i) −*x*+1, *y*−1/2, −*z*+1/2; (ii) *x*+1, *y*, *z*.

## References

[bb1] Bruker (1997). *SMART* and *SAINT* Bruker AXS Inc., Madison, Wisconsin, USA.

[bb2] Li, Y.-F. & Jian, F.-F. (2010). *Acta Cryst.* E**66**, o2061. 10.1107/S1600536810027959 PMC3007564 21588365

[bb3] Sheldrick, G. M. (2008). *Acta Cryst.* A**64**, 112–122. 10.1107/S0108767307043930 18156677

